# Creatine Protects against Excitoxicity in an In Vitro Model of Neurodegeneration

**DOI:** 10.1371/journal.pone.0030554

**Published:** 2012-02-08

**Authors:** Just Genius, Johanna Geiger, Andreas Bender, Hans-Jürgen Möller, Thomas Klopstock, Dan Rujescu

**Affiliations:** 1 Department of Psychiatry, Ludwig-Maximilians-University, Munich, Germany; 2 Department of Neurology, Ludwig-Maximilians-University, Munich, Germany; Roswell Park Cancer Institute, United States of America

## Abstract

Creatine has been shown to be neuroprotective in aging, neurodegenerative conditions and brain injury. As a common molecular background, oxidative stress and disturbed cellular energy homeostasis are key aspects in these conditions. Moreover, in a recent report we could demonstrate a life-enhancing and health-promoting potential of creatine in rodents, mainly due to its neuroprotective action. In order to investigate the underlying pharmacology mediating these mainly neuroprotective properties of creatine, cultured primary embryonal hippocampal and cortical cells were challenged with glutamate or H_2_O_2_. In good agreement with our *in vivo* data, creatine mediated a direct effect on the bioenergetic balance, leading to an enhanced cellular energy charge, thereby acting as a neuroprotectant. Moreover, creatine effectively antagonized the H_2_O_2_-induced ATP depletion and the excitotoxic response towards glutamate, while not directly acting as an antioxidant. Additionally, creatine mediated a direct inhibitory action on the NMDA receptor-mediated calcium response, which initiates the excitotoxic cascade. Even excessive concentrations of creatine had no neurotoxic effects, so that high-dose creatine supplementation as a health-promoting agent in specific pathological situations or as a primary prophylactic compound in risk populations seems feasible. In conclusion, we were able to demonstrate that the protective potential of creatine was primarily mediated by its impact on cellular energy metabolism and NMDA receptor function, along with reduced glutamate spillover, oxidative stress and subsequent excitotoxicity.

## Introduction

The protective potential of creatine (1-methyl-guanidino acetic acid) has been extensively assessed in various models of neurodegeneration, including *in vivo* models of oxidative stress [1], [2].

Aging, neurodegenerative diseases like Alzheimer's disease, Huntington's disease and amyotrophic lateral sclerosis, and potentially also neuropsychiatric disorders like schizophrenia share some bioenergetic core features, specifically the contribution of oxidative stress caused by a progressive dysfunction of the respiratory chain along with mitochondrial DNA damage [3]–[5]. Thus, as a potential antioxidative agent and buffer of intracellular energy stores, creatine - specifically in a preventive approach - may also become an interesting new agent to increase life span and to delay the progression of the disorders mentioned above.

In neuronal cells, aerobic glycolysis is the primary source for ATP synthesis [6]. As stores of glucose, glycogen and O_2_ are limited in the brain, the availability of the creatine kinase/phosphocreatine (CK/PCr) system may operate as an important alternative energy source in tissues or subcellular compartments with high and fluctuating energy demands, e.g. in neurons [7]. Based on substrate level phosphorylation of adenine with CK/PCr this system is capable of rapidly restoring ATP levels within certain limits, determined by the tissue concentrations of creatine/CPK itself and the enzymatic system required for phosphorylation and phosphate group transfer. ATP is required to maintain the function of energy-demanding Na^+^/K^+^-ATPase and Ca^2+^-ATPase, thus preserving the membrane potential [8]. Considering that high relative CK activity could be demonstrated in the brain [9], it has been concluded that this enzyme serves as a key factor in the CNS energy metabolism. In support of this notion, a direct correlation between CK flux and brain activity has been provided by *in vivo* **^31^**P nuclear magnetic resonance transfer determinations [10], [11]. The brain-specific isoform of the CK (CK-BB) in concert with a mitochondrial isoform (uMT-CK) and the required substrates (creatine/PCr) regulate intracellular ATP levels [12]. Via formation of an CK “energy shuttle”, CK activity has moreover been discussed to be directly implicated in neurotransmitter release, maintainance of membrane potentials and restoration of ion gradients over the membrane after depolarization [12]–[14].

Primarily, creatine is synthesized in a two step mechanism via AGAT (arginine: glycine amidinotransferase) in the kidney and pancreas [15]. The resultant guanidinoacetate is then shuttled to the liver, where it is subsequently methylated by GAMT (guanidinoacetate methyltransferase to result in creatine which ultimately is actively exported to tissues where it is energetically required. Loss of GAMT activity results in a well-defined creatine deficiency syndrome, which is characterized by developmental delay, neurological dysfunction and mental retardation [16]. In Huntington's disease, a further neurodegenerative condition, brain-type creatine kinase expression is reduced, which might contribute to damage in specifically energy-demanding tissues such as the brain and the cochlea, where intact energy shuttling processes are crucial [17]. The endogenous *de novo* creatine synthetic activity in the brain is rather low. It is interesting to note, that GAMT was identified to act as a novel target for p53, which serves as a further mechanism for metabolic stress adaptation [18]. Under normal conditions dietary intake constitutes about 50% of the total creatine content of the organism. Moreover, the blood-brain barrier permits passage of systemically supplemented creatine to the brain [19], which ultimately reaches the neuronal cytoplasm via a specific sodium and chloride dependent transmembrane transporter (CRT) working against a concentration gradient [20]. We thus speculate, that a specific diet should serve as an efficient strategy to enhance brain tissue creatine concentrations and establish an “energy buffer”.

In a previous report, we demonstrated that creatine supplementation in mice could increase healthy life span. Beyond a moderately increased life span, the most favourable effects of creatine related to neurobehavioral performance, most markedly in memory tests [21]. In an attempt to gain a better understanding of these neuroprotective properties on the cellular level, we conducted a study on a hippocampal cell culture model.

## Materials and Methods

### Hippocampal embryonal cell culture

Pregnant Long Evans rats (Janvier Breeding Centre, Le Genest Saint Isle, France) were decapitated under deep CO_2_ anaesthesia. The embryos (embryonic day 17/18) were rapidly microdissected on ice and the hippocampal tissue was dissociated by mechanical homogenization in a Hank's balanced salt solution (HBSS) without Ca^2+^ and Mg^2+^ buffered with 10 mM HEPES at pH 7.4 and supplemented with 1 mM sodium pyruvate and 4% bovine serum albumin. The tissue was digested with a HBSS solution containing 2 mg/ml papain and 1000 kU/ml DNAse I. Debris was removed by two steps of centrifugation at 800 g for 15 min each. The resulting cell pellet was resuspended by gentle trituration through a blue polysterene pipet tip. The live (dye-exluding) purified cells were counted in a hematocytometer by mixing 20 µl of the suspension with 20 µl of 0.4% trypan blue solution, plated at a density of 0.8×10^5^ cells/48 well plate and cultivated in a defined medium (Neurobasal with antioxidant-free B27 supplement and 0.5 mM glutamine, 50 µg/ml gentamycin, GIBCO BRL, Life Technologies Ltd, Paisley, UK) on L-ornithine-coated tissue culture dishes (Nalge Nunc International, Rochester, NY, USA) at 95% air, 5% CO_2_ in a humidified incubator. Every 72 h and immediately preceding the experiment one half of the medium volume was replaced by fresh medium. Experiments were performed on 15–17 DIV (days *in vitro*). Cell culture quality was routinely assessed by viability analyses, morphological parameters and immunostaining for neuronal and glial cell markers. Glial cells identified by GFAP immunofluorescence represented <1% of the total cell population, while >99% of the cells expressed NeuN and β-3-tubulin (TUJ-1) as neuronal markers.

Experiments were performed in accordance with the German law on the use of animals and approved by the local bioethical committee.

### Intracellular Ca^2+^ measurements

Briefly, cells (1×10^6^/ml) in calcium buffer (140 mM NaCl, 5 mM KCl, 0.5 mM MgCl_2_, 20 mM HEPES, 1 mM CaCl_2_, 10 mM glucose) were loaded with the “leakage-resistant” dye Fura PE3-AM (1.25 µM) at 37°C for 40 minutes probenecid to reduce compartmentalization and dye leakage). Immediately prior to measurement, after 30 minutes for intracellular deesterification cells were transferred to a Perkin-Elmer L50B spectrofluorimeter equipped with a temperature controlled cuvette chamber and allowed to equilibrate to 37°C while gently stirring. Experiments were started after obtaining stable fluorescence ratios (R) under dual wavelength excitation (340/380 nm) with a 500 nm emission cut-off wavelength for at least 3 minutes. Stimulatory agents or DMSO vehicle were injected directly in the sample. Correction for autofluorescence was performed by parallel processing of DMSO blank samples. For *in situ* calibration of Fura PE 3 fluorescence after each experiment cells were treated with with 10 µM ionomycin and Rmax was measured. EGTA (5 mM, 30 mM Tris, pH 8.5) was subsequently added to obtain Rmin. [Ca^2+^]_i_ was then calculated using the Grynkiewicz equation [22].

### LDH assay

Cell death was assessed by determination of the lactate dehydrogenase (LDH) leakage from the damaged cells into the medium supernatant after different time intervals following treatment using a commercially available kit (CytoTox 96, Promega, Madison, WI, USA).

### Glutamate determination

L-Glutamate was measured by an enzymatic assay according to the supplier's instructions (Amplex Red™ Glutamate assay kit, Molecular Probes, Eugene, Oregon, USA). Protein concentration was determined by the Bradford assay (Biorad, Munich, Germany).

### ATP/Phosphocreatine assay

ATP and phosphocreatine as markers of the cellular energy charge were determined by luciferin-luciferase chemiluminescence in cell lysates (CellTiter-Glo Luminescent Cell Viability Assay, Promega, Madison, Wisconsin, USA). Protein concentrations, determined by the Bradford assay were taken as a reference.

### Cell-free chemiluminescent determination of superoxide scavenging by creatine

Xanthine oxidase (0.025 U/ml) and xanthine (100 µM) were incubated in PBS in order to yield a continuous superoxide generator. After addition of lucigenin (bis-N-methylacridiniumnitrate, 50 µM final concentration) and occurrence of stable chemiluminescence (CL) signals creatine at rising concentrations was added to the system and CL was recorded in a tube luminometer. Background CL was simultaneously determined and subtracted. The specificity of CL for stimulated O_2_^−^ release was verified by adding superoxide dismutase (SOD), the cell-permeable SOD mimic MnTBAP (manganese[III]tetrakis[4-benzoic acid]porphyrin), or the low molecular weight O_2_^−^ scavenger tiron (4,5-dihydroxy-1,3-benzene-disulfonic acid).

### Statistical analysis

If not otherwise specified, data were analyzed with the SPSS software version 14.0 (SPSS Inc., Chicago, IL, USA). For statistical analysis either Student's t-test or one-way ANOVA followed by Kruskal-Wallis *post hoc* test was used were appropriate. Data are expressed as means +/− SD in normally distributed data. P values of <0.05 were considered as statistically significant using a two-tailed estimation.

## Results

### Creatine does not act as an antioxidant

The antioxidant properties of creatine as a superoxide scavenger were tested in a cell-free environment employing xanthine oxidase/xanthine as an enzymatic generator of superoxide anions. In this system, creatine added in concentrations up to 5 mM did not reveal any antioxidant properties. In contrast, a rise of chemiluminescence was seen after adding creatine, indicating increased superoxide generation or enhanced life-time of these species (105.2+/−3.1% of control, p = 0.009).

### Creatine incubation for extended periods does not induce cytotoxicity

Physiological creatine levels in the CNS are settled in the range from 10–30 µmol/g wet weight. In our experiments no overt signs of neurotoxicity like cell detachment, alterations of cellular shape or retraction of cellular processes could be observed at concentrations ranging up to 10 mM, even if extending the incubation period for up to 5 days.

### Creatine mediates neuroprotection against excitotoxicity

**Figure 1:**
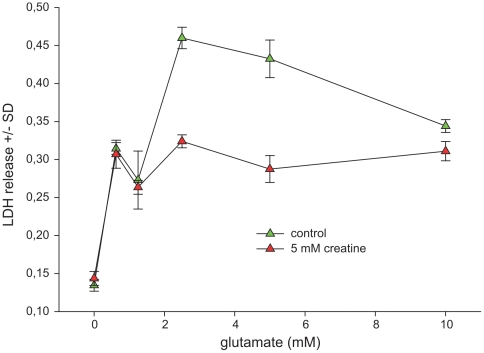
Protective effect of creatine in hippocampal cell cultures exposed to glutamate. Hippocampal cells (DIV 17) were incubated with rising concentrations of glutamate in absence or in presence of 5 mM creatine. After 24 h the LDH release into the cell culture supernatant was determined. Total protein of the lysed cell monolayer was used as a reference. Data are expressed as arbitrary units per mg protein +/− standard deviation. Each data point represents the mean of triplicates. Each experiment was independently performed in triplicate. Statistical analysis was performed by unpaired Student's T-test. *denotes statistical significance at a level of p<0.01.

LDH leakage as a marker of cytotoxicity was dose-dependently increased under glutamate challenge, along with morphological alterations including retraction of axonal/dendritic processes and detachment from the cell culture dishes. Toxicity was substantially mitigated in cell cultures co-incubated with creatine at 5 mM concentration, even in the glutamate high-dose range. Under baseline conditions (no glutamate challenge) cell viability in hippocampal cultures was not significantly enhanced (Fig. 1).

### Creatine enhances the cellular energy charge

**Figure 2:**
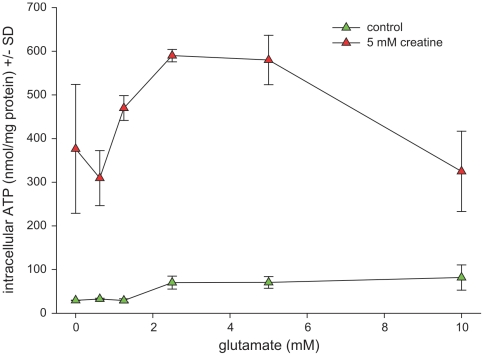
Effect of creatine on intracellular ATP/Phosphocreatine content in hippocampal cells exposed to glutamate. Hippocampal cells (DIV 17) were challenged with glutamate at rising concentrations in absence or presence of 5 mM creatine. After 24 h of incubation the cells were harvested and intracellular ATP/PCr concentration was determined by luciferin/luciferase chemiluminescence. Total protein content of the cell lysate was employed as a reference. Data are expressed as intracellular ATP concentration equivalents corrected for total protein +/− standard deviation. Each data point represents the mean of triplicates. The experiment was independently performed in triplicate. Unpaired Student's T-test was used for statistics. *denotes statistical significance at a level of p<0.01.

Hippocampal cells having been incubated with creatine contained substantially higher concentrations of ATP/Phosphocreatine determined under baseline conditions than control cell cultures. Thus the bioenergetic utilization of creatine was extremely efficient. Unexpectedly, glutamate concentrations, if not exceeding 5 mM, did not yield energy depletion but rather led to enhanced intracellular ATP/phosphocreatine levels This phenomenon was most pronounced in creatine-supplemented cells (Fig. 2).

### Creatine prevents glutamate spillover but fails to mediate neuroprotection against experimentally induced oxidative stress

**Figure 3:**
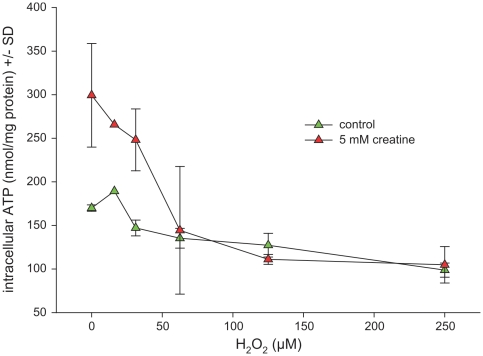
Effect of creatine on intracellular ATP/Phosphocreatine content in hippocampal cells under oxidative stress. Hippocampal cells (DIV 15) were challenged with hydrogen peroxide at rising concentrations in absence or presence of 5 mM creatine. After 24 h the cells were harvested for determination of intracellular ATP/PCr concentration, which was determined by luciferin/luciferase chemiluminescence and for measurement of total protein content, which served as a reference. Data are expressed as intracellular ATP concentration equivalents corrected for total protein +/− standard deviation. Each data point represents the mean of triplicates. The experiment was independently performed in triplicate. Unpaired Student's T-test was used for statistics. *denotes statistical significance at a level of p<0.01.

**Figure 4:**
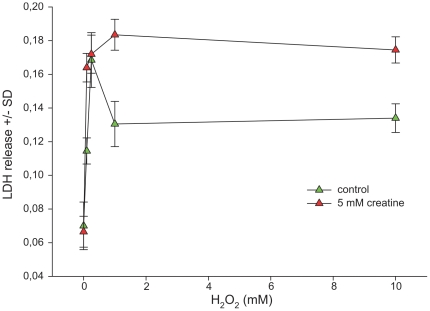
Protective effect of creatine in hippocampal cell cultures challenged with oxidative stress. Hippocampal cells (DIV 15) were incubated with hydrogen peroxide in rising concentrations in absence or in presence of 5 mM creatine. After 24 h the LDH release into the cell culture supernatant was assessed. Total protein of the cell monolayer was used as a reference. Data are expressed as arbitrary units per mg protein +/− standard deviation. Each data point represents the mean of triplicates. Each experiment was independently performed in triplicate. Statistical analysis was performed by unpaired Student's T-test. *denotes statistical significance at a level of p<0.01.

**Figure 5:**
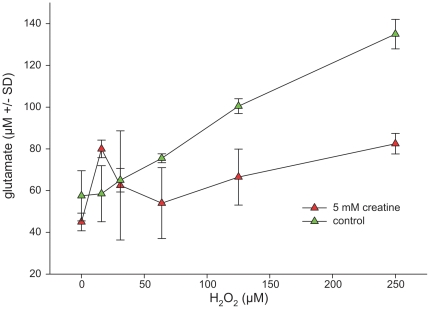
Impact of creatine on glutamate efflux into the supernatant in hippocampal cell cultures exposed to hydrogen peroxide. Hippocampal cells (DIV 15) were incubated with rising concentrations of hydrogen peroxide in absence or in presence of 5 mM creatine. After 24 h the glutamate release into the cell culture supernatant was enzymatically determined. Total protein of the lysed cell monolayer was used as a reference. Data are expressed as glutamate concentration per mg protein +/− standard deviation. Each data point represents the mean of triplicates. Each experiment was independently performed in triplicate. Statistical analysis was performed by unpaired Student's T-test. *denotes statistical significance at a level of p<0.01.

Hydrogen peroxide (H_2_O_2_) was added to the cell culture supernatant to induce oxidative stress. This condition led to a depletion of intracellular energy levels after 18 h of incubation (Fig. 3), along with enhanced LDH release into the supernatant (Fig. 4). Creatine at a concentration of 5 mM applied 3 h before H_2_O_2_ was added could maintain enhanced intracellular ATP/phosphocreatine concentrations as long as H_2_O_2_ concentrations remained well below 60 µM. Beyond this concentration energy levels were not altered by creatine pretreatment (Fig. 3). Unexpectedly, creatine aggravated H_2_O_2_-induced toxicity at high H_2_O_2_ concentrations and failed to reduce LDH release going along with H_2_O_2_ exposure, even at low concentrations (Fig. 4). In contrast, extracellular glutamate concentrations reflecting an overflow (and secondary hyperexcitability) which occurs along with oxidative stress were effectively reduced following creatine incubation (Fig. 5). Thus, creatine seems to efficiently interfere with this vicious circle which maintains the excitotoxic cascade *after* its initiation. Even under non-stressful baseline conditions glutamate concentrations remained reduced in creatine-treated hippocampal cell cultures. These effects were far less pronounced in mixed cortical cell cultures (data not shown). As glial cells were almost absent in our model the popular explanation for the H_2_O_2_ induced glutamate excess as an inhibition of redox-sensitive glutamate transporters leading to secondary pathology [23] seems to reflect only one partial aspect of the molecular mechanisms. The discrepancy between stabilization against secondary glutamate spillover and enhanced H_2_O_2_ toxicity in presence of creatine remains to be investigated. We tend to speculate that H_2_O_2_ neurotoxicity is not always necessarily due to the secondary glutamate excess, which was efficiently antagonized here.

### Creatine attenuates the Ca^2+^ response following NMDA receptor stimulation

**Figure 6:**
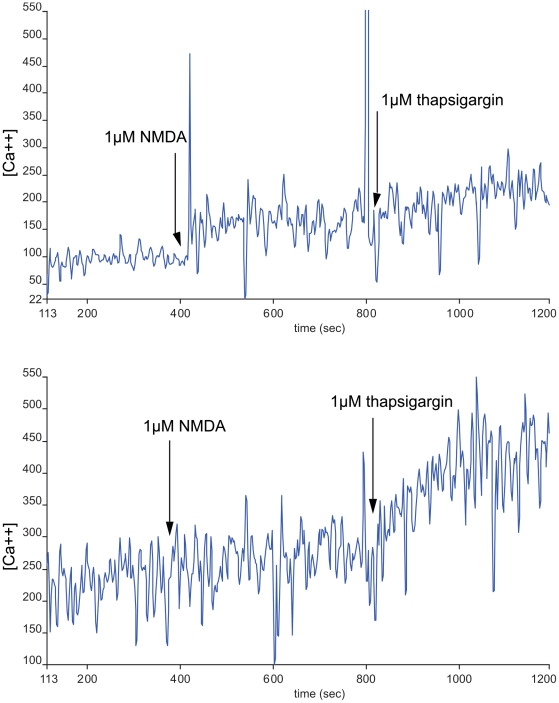
Impact of creatine pre-incubation on NMDA-triggered intracellular calcium rise in hippocampal cells. Hippocampal cell cultures (DIV 18) were incubated with 5 mM of creatine for 18 h. Cells were harvested, dissociated and loaded with FURA PE-3/AM. Ca^2+^ ratiometry was performed in 0.5×10^6^ cells/ml at 37°C. After stable baseline ratios were achieved NMDA was added and the response was recorded for 400 seconds. Thapsigargin was added for SERCA inhibition. The tracings are representative for 5 individual experiments by calculating curve means. Data for intracellular Ca^2+^ are expressed in arbitrary units. The second tracing shows responses in creatine-pretreated cells, the first one has been acquired from control cells.

Following 18 h preincubation with 5 mM creatine (which yielded no significant toxicity) and careful washout, the rise of intracellular Ca^2+^ ions in response to NMDA receptor stimulation at supramaximal doses (1 µM) was almost completely abolished, while in a non-receptor mediated control experiment the response to addition of the SERCA (sarcoplasmic/endoplasmic reticulum calcium ATPase) inhibitor thapsigargin (500 nM), which leads to a depletion of intracellular Ca^2+^ stores was largely preserved (Fig. 6).

## Discussion

Depletion of high-energy phosphates, such as ATP and phosphocreatine (PCr) is an early event in the neurotoxicity of glutamate [24]–[27]. Abnormal calcium uptake into mitochondria has also been reported following exposure to glutamate [28]–[29]. Profound disruption of the cellular energy ultimately leads to a decreased GTP concentration [30] along with altered activity of GTP binding proteins, such as Rac and Ras, which yields a proapoptotic state [31]. Juravleva et al. [32] hypothesized that the maintainance of the cellular energy charge by creatine may shift the apoptotic balance towards the Ras-mediated antiapoptotic PI3K/AKT or survival signal pathways (PI3K/Rac/NAD(P)H-oxidase/ROS/NF-kappaB), specifically the Ras/NFkappaB system, where multiple pathways mediating survival converge via stabilization of GTP levels. Indeed, creatine was shown to maintain neuronal/glial survival following glutamate treatment, which correlated with decreased levels of farnesylated Ras and the NF-kappaB inhibitor IkappaB and *increased* levels of ROI [32].

Extending our *in vivo* data on creatine as a neuroprotective and life-enhancing agent [21], we designed a series of *in vitro* experiments in order to dissect the underlying pharmacology. For biochemical analysis, cell culture models carry the advantage of a reduced biological complexity. This does especially apply to the case of creatine metabolism. Here, on account of systemic sources and a complex pharmacokinetics through various body compartments it would be almost impossible to establish controlled conditions of creatine supply in a specific concentration range *in vivo*. Expression of BB-CK and uMt-CK has previously been demonstrated to occur as early as embryonic day 14, along with significant CK activity [33]. Thus the cell culture model we chose seems suitable to assess the neuroprotective potential of creatine. As GFAP and NeuN staining revealed that >99% of all cells in the cultures were neurons, a significant contribution of potential glial cells to the biochemical effects in response to creatine is highly improbable.

Generally speaking, our findings are in line with previous *in vitro* studies on neuroblastoma, hippocampal and mixed cerebrocortical cell cultures, which all have shown the potential of creatine to prevent glutamate-induced neurotoxicity [7], [32]. Still, the underlying mechanisms, specifically with reference to a potential interference with ROI generation as a downstream event in the excitotoxic pathway remained amazingly elusive.

Moreover, it remained to be clarified, how creatine may interfere with the glutamate metabolism on a cellular level. It has been hypothesized that these effects are mediated by supporting mechanisms involved in the glutamate-glutamine cycle, an activity with a demand of about 60–80% of the energy derived from glucose metabolism [34]: Glial glutamate uptake from the synaptic cleft is primarily performed by GLT-1 [35]. Glutamine synthetase and glutaminase involved in glutamine transport in the presynaptic neuron [36] or oxidation to 2-oxo-glutarate, which enters the citric acid cycle [36]–[38] are ATP-dependent, likewise. In our rodent creatine supplementation study, gene expression analysis revealed an almost twofold upregulation of the high affinity glutamate transporter Slc1a3, which should also accelerate the clearance of excessive extracellular glutamate [21]. It may also be speculated, that nutrient-sensing pathways, such as mTOR (Target of Rapamycin) and thereby cell proliferation and senescence might be directly or indirectly regulated by creatine [39].

Interestingly, although such glial–cell mediated mechanisms were practically absent in our cell culture model, we could deliver direct evidence for a massively improved supply of ATP-bound energy in isolated hippocampal cells. Not unexpectedly, under these conditions cells became more resistant to withstand an excitotoxic challenge with glutamate.

The antioxidant properties of creatine remain another controversial issue: It is generally maintained that glutamate toxicity is essentially associated with the excessive generation of reactive oxygen species as a downstream event, eventually leading to macromolecule alterations and cytotoxicity. The data on antioxidant properties of creatine is somewhat controversial: Lawler et al. [40] were able to deliver evidence for a direct antioxidant potential [40], a view other authors and ourselves cannot share: Unexpectedly, performing spin-trapping EPR spectroscopy, Juravleva et al. [32] demonstrated an *augmented* EPR superoxide signal when creatine was added to glutamate-treated cortical/glial cell cultures [32]. We were able to reproduce these findings in a cell-free environment employing xanthine oxidase/xanthine as an enzymatic superoxide generator. Here, creatine tested in a range up to 5 mM did not reveal any antioxidant properties, but rather led to a slightly *enhanced* chemiluminescence reflecting increased superoxide generation or enhanced life-time of these species. These observations correspond well with our own in vitro data suggesting an enhanced cytotoxicity of H_2_O_2_ in presence of creatine.

Paradoxically and in contrast to our own data, the above mentioned authors could observe *better* cell viability under these conditions, drawing the conclusion that the enhanced generation of oxygen radicals may constitute a decisive factor for the activation of redox-dependent survival pathways. Interestingly, in some of our cell preparations H_2_O_2_ at very low levels (low micromolar range) seemed to *support* hippocampal cell viability (data not shown).

To our knowledge, our report is the first to deliver evidence for a direct interference of creatine with the NMDA-receptor mediated neurotransmission. We were able to demonstrate that creatine pre-treatment leads to a substantially reduced Ca^2+^ response to NMDA. It should be noted that these observations were made after a thorough creatine washout. Therefore, permanent alterations of the NMDA receptor must have taken place throughout the incubation period.

Altogether the anti-aging and neuroprotective effect of creatine seems to result from multiple single effects, ranging from an economization of the cellular energy metabolism up to not yet completely understood antiexcitotoxic effects taking place on the receptor level or subsequent Ca^2+^ mediated pathways, a phenomenon which deserves further investigation. Although widely postulated, we found no direct evidence for a direct antioxidative action of creatine.
